# A Collaborative Approach to Identifying Social Media Markers of Schizophrenia by Employing Machine Learning and Clinical Appraisals

**DOI:** 10.2196/jmir.7956

**Published:** 2017-08-14

**Authors:** Michael L Birnbaum, Sindhu Kiranmai Ernala, Asra F Rizvi, Munmun De Choudhury, John M Kane

**Affiliations:** ^1^ The Zucker Hillside Hospital Northwell Health Glen Oaks, NY United States; ^2^ Feinstein Institute of Medical Research Manhasset, NY United States; ^3^ Hofstra Northwell School of Medicine Hempstead, NY United States; ^4^ Georgia Institute of Technology Atlanta, GA United States

**Keywords:** schizophrenia, psychotic disorders, online social networks, machine learning, linguistic analysis, Twitter

## Abstract

**Background:**

Linguistic analysis of publicly available Twitter feeds have achieved success in differentiating individuals who self-disclose online as having schizophrenia from healthy controls. To date, limited efforts have included expert input to evaluate the authenticity of diagnostic self-disclosures.

**Objective:**

This study aims to move from noisy self-reports of schizophrenia on social media to more accurate identification of diagnoses by exploring a human-machine partnered approach, wherein computational linguistic analysis of shared content is combined with clinical appraisals.

**Methods:**

Twitter timeline data, extracted from 671 users with self-disclosed diagnoses of schizophrenia, was appraised for authenticity by expert clinicians. Data from disclosures deemed true were used to build a classifier aiming to distinguish users with schizophrenia from healthy controls. Results from the classifier were compared to expert appraisals on new, unseen Twitter users.

**Results:**

Significant linguistic differences were identified in the schizophrenia group including greater use of interpersonal pronouns (*P*<.001), decreased emphasis on friendship (*P*<.001), and greater emphasis on biological processes (*P*<.001). The resulting classifier distinguished users with disclosures of schizophrenia deemed genuine from control users with a mean accuracy of 88% using linguistic data alone. Compared to clinicians on new, unseen users, the classifier’s precision, recall, and accuracy measures were 0.27, 0.77, and 0.59, respectively.

**Conclusions:**

These data reinforce the need for ongoing collaborations integrating expertise from multiple fields to strengthen our ability to accurately identify and effectively engage individuals with mental illness online. These collaborations are crucial to overcome some of mental illnesses’ biggest challenges by using digital technology.

## Introduction

Social media provides an unprecedented opportunity to transform early psychosis intervention strategies, especially for youth who are both the highest utilizers of social media and at the greatest risk for the emergence of a psychotic disorder. Social media, defined as any form of online communication through which users create virtual communities to exchange information, ideas, messages, pictures, and videos, has forever changed the way youth interact, learn, and communicate. More than 90% of US youth use social media daily [1], placing it ahead of texting, email, and instant messaging, and they disclose considerably more about themselves online than offline [2]. Globally more than 2 billion users engage with social media regularly [3] and Twitter represents one of the most popular platforms with over 300 million monthly users worldwide.

Individuals with mental illness similarly report regularly engaging with social media [4]. Identified benefits include developing a sense of belonging, establishing and maintaining relationships, accessing support, challenging stigma, raising awareness, and sharing experiences [4,5]. Youth with newly diagnosed schizophrenia in particular report frequently utilizing social networking sites throughout the course of illness development and treatment, engaging in social media activity several times daily, and spending several hours per day online [6].

Harvesting social media activity has become an established source for capturing personalized and population data in the forms of explicit commentary, patterns and frequency of use, as well as in the intricacies of language. The massive amount of data available online has been accompanied by major advancements in computational techniques capable of quantifying language and behavior into statistically meaningful measures. There is now clear and convincing evidence that online activity can be used to reliably monitor and predict health-related behaviors [7] ranging from the spread of the influenza virus across the United States to rates of seasonal allergies, HIV infection, cancer, smoking, and obesity [8-10].

The most robust data source available is made up of the words users post online. Prior work in speech and text analysis has identified reliable linguistic markers associated with schizophrenia, including significant differences in word frequency, word categories, and use of self-referential pronouns [11-15]. These same language analytic tools have been successfully implemented to analyze modern social media-based communication [16] and have demonstrated significant linguistic differences in posts written by individuals with schizophrenia compared to individuals with depression, physical illness, and healthy controls [17]. Furthermore, classifiers designed to automatically sort individual cases into diagnostic categories have achieved success in recognizing participants with psychotic disorders from healthy controls based on linguistic differences in writing samples [15] and speech [13,18].

Researchers have begun to build classifiers aiming to identify individuals online who may have schizophrenia *without* a confirmed clinical diagnosis by scanning publicly available Twitter feeds for self-disclosures. Language-based computational models have achieved more than 80% and 90% accuracy [19,20] in correctly identifying users with self-reported schizophrenia from healthy controls. Unfortunately, however, it is challenging to confirm the authenticity of online self-disclosures. Furthermore, prior work has demonstrated that words that might have been automatically identified as self-disclosure such as “psychosis,” schizophrenia,” and “delusion” are often used inappropriately online [21] and may represent a major limitation to these computational models. To date, limited efforts have involved expert input to evaluate the authenticity of diagnostic self-disclosures.

To move from noisy diagnostic inferences to accurate identification, we propose a human-machine partnered approach, wherein linguistic analysis of content shared on social media is combined with clinical appraisals. This project aims to explore the utility of social media as a viable diagnostic tool in identifying individuals with schizophrenia.

## Methods

Initial data acquisition involved extracting publicly available Twitter posts from users with self-disclosed diagnoses of schizophrenia. Case-insensitive examples include “I am diagnosed with schizophrenia,” “told me I have schizophrenia,” and “I was diagnosed with schizoaffective disorder” (Textbox 1). Prior work identifying markers of mental illness online used similar filtering techniques based on self-reported diagnoses [22,23]. Data were extracted from Twitter because posts are often publicly accessible and readily available for analysis by researchers. Approval from the institutional review board was not sought because these data were freely available in the public domain and researchers had no interaction with the users.

These search queries resulted in 21,254 posts by 15,504 users between 2012 and 2016. For each user, Twitter timeline data from 2012 to 2016 were collected using a Web-based Twitter crawler called GetOldTweetsAPI [24], which scrapes public Twitter profiles to obtain historical Twitter data in a structured format. The data included tweet text, username, posting time, hashtags, mentions, favorites, geolocation, and tweet ID. A subsample of 671 users from the primary dataset was randomly selected (each user had equal probability of being selected) and provided to two clinicians for appraisal. As a control group, a random sample of Twitter users was collected from individuals without any mentions of “schizophrenia” or “psychosis” in their timeline. Descriptive statistics of the acquired data are shown in Table 1.

**Search queries for Twitter data collection.**

- Diagnosed me with (schizophrenia | psychosis)
- Diagnosed schizophrenic
- I am diagnosed with (psychosis | schizophrenia)
- I am schizophrenic
- I have been diagnosed with (psychosis | schizophrenia)
- I have (psychosis | schizoaffective disorder | schizophrenia)
- I think I have schizophrenia
- My schizophrenia
- They told me I have schizophrenia
- I was diagnosed with (psychosis | schizoaffective disorder | schizophrenia)
- Told me I have (psychosis | schizophrenia)

**Table 1:** Descriptive statistics of acquired Twitter data.

Results	Schizophrenia group (n=146)	Control group (n=146)
Total tweets by unique users, n	1,940,921	791,092
Mean tweets per user, mean (SD)	13,293.93 (18,134.83)	5418.43 (11,403.54)
Median tweets per user, median (IQR)	5542.5 (14,651.8)	1660.0 (4402.3)
Range of tweets per user (min-max)	8-88,169	1-82,985

### Clinician Appraisal

To eliminate noisy data (disingenuous, inappropriate statements, jokes, and quotes) and obtain a cleaner sample of schizophrenia disclosures likely to be genuine, a psychiatrist and a graduate-level mental health clinician (authors MB and AR) from Northwell Health’s Early Treatment Program, with extensive expertise in early stage schizophrenia, annotated the data. For each user, their disclosure tweet and the 10 consecutive tweets before and after were extracted to assist in making an authenticity determination. Each user was annotated by categorizing them into one of three classes. Class “yes” contained users who appeared to have genuine disclosures. Class “no” contained users who had inauthentic posts, including jokes, quotes, or were from accounts held by health-related blogs. Class “maybe” contained users for whom the experts could not confidently appraise the authenticity of the disclosure (Textbox 2). Each clinician first categorized users separately and subsequently reviewed findings together to achieve consensus. Interrater reliability for classes “yes” and “no” was 0.81 (Cohen kappa). Disagreement arose on ambiguous disclosure statements. Clinicians then utilized additional input from surrounding tweets to make an authenticity determination. These users were most often annotated as “maybe.” The annotation task for 671 users resulted in 146 yes, 101 maybe, and 424 no users. These three classes of users shared 1,940,921, 1,501,838, and 8,829,775 tweets, respectively, with a mean (SD) of 13,293.98 (18,134.83), 14,869.68 (19,245.88), and 20,824.94 (45,098.07) tweets per user.

### Classification Method

#### Data Preparation

To distinguish users with disclosures deemed genuine from the regular Twitter stream, the problem was modeled as a machine learning classification task. Users who had been annotated with class yes, formed the positive examples (class 1) for the classifier. A sample of same size collected from the control group formed the negative examples (class 0). Given the ambiguity of the “maybe” class, it was left out of this initial model. The training dataset, constructed by combining both positive and negative examples resulted in 292 users. The classifier was built and evaluated by applying 10-fold cross-validation, an established technique in supervised machine learning [25].

#### Classification Framework

Using the training datasets described previously, a supervised learning framework was used to build the classifier. The classification framework involved three steps: featurizing training data, feature selection to improve predictive power, and classification algorithm.

##### Featurizing Training Data

The textual data from Twitter timelines was used to generate features for the classifier. Each tweet in the user’s timeline was represented using the following features:

**Examples of tweets annotated as “yes,” “no,” and “maybe.”**

**Annotated “yes”**

- MY MOM TOOK ME TO THE FUCKING DOCTOR AND MY DOCTOR TOLD ME I HAVE SCHIZOPHRENIA
- Finally home, was in a mental hospital for the last eight days:/ I found out I have schizophrenia...
- My parents and sister are the only family that know about my schizophrenia & everyones talking bad about it
- i have schizophrenia im bound to a life in psych wards hearing voices
- Welcome to crazy town. I figure the best way to tell the family I have psychosis is to take a picture of all my meds post it on fb with the tag of its official”
- Today was basically hell. I had to bullshit my way through it pretending like I was fine with my schizophrenia flaring up again. Urgh.
- I’ll give you my Risperdal. it’s my old med to treat my schizophrenia, I took it once and I slept for 12 hours
- I have schizophrenia/depression. I am trying to become better by exercise and working I have a job xoxo I love Saturday xx
- I watched your video about depression. I have schizophrenia, epilepsy and depression. I am very proactive although. :)
- And it frightens me to say that I know you don’t picture me when you imagine a schizophrenic, even although I’m likely the only one you know.

**Annotated “no”**

- Twitter is basically an acceptable way to talk to yourself w/o being diagnosed schizophrenic
- Decided to practice my speech at the union. To the naked eye I’m sure it just looks like I have schizophrenia
- My schizophrenia article got approved for my #Psychopharmacology presentation! #yass #cantstopwontstop
- Sometimes I wish I have schizophrenia. So I can escape the reality.
- I always talk about myself as if I have schizophrenia. You gonna do this thing Aidan?” “I don’t know. I doubt that I’m going to do that”“
- Roses are red Violets are blue I am schizophrenic And so am I
- Texas inmate set to die, but lawyers say he’s delusional: Diagnosed schizophrenic killed his in-laws
- She loves my schizophrenia, it embraces every side of me.
- Could schizophrenia simply be an extremely spiritually sensitive person, surrounded by crazy-makers? I think so.
- Watching True Life: I Have Schizophrenia Yessss... My kinda topic, future Clinical Psychologist right here!

**Annotated “maybe”**

- I am thoroughly convinced that my schizophrenia is a better friend than you.
- Yes, I have schizophrenia. No, I am not crazy.
- Seven days, my schizophrenia breaks-my brain waves distorted. theyre going in the trunk to avoid detection”
- is it my schizophrenia? I always knew it was...
- oh no. (To future employers) it’s my schizophrenia
- it’s me. I’m the inconsistent lady and i have schizophrenia
- ran up with a shovel. wonder if she felt bad afterwards. I would probably be like sorry it was my schizophrenia
- OMG U R SO FUNNY!1!!!!1!!!!!”it’s just my schizophrenia
- can’t help it my schizophrenia is hard to contain
- must stop listening to the talking cake, must stop listening to the talking cake, where’s my schizophrenia medication

*n*-Gram language model: a language model of 500 top unigrams, bigrams, and trigrams (ie, sequences of one, two, and three words) was generated from the entire timeline data of all users. Each tweet was represented as a feature vector of normalized term frequency-inverse document frequency (tf-idf) frequency counts of the top 500 *n*-grams.

Linguistic inquiry and word count (LIWC): The widely validated LIWC lexicon [26] was employed, which identifies linguistic measures for the following psycholinguistic categories: (1) affective attributes, including positive and negative affect, anger, anxiety, sadness, swearing; (2) cognitive attributes, including both cognition categories comprising of cognitive mechanisms, discrepancies, inhibition, negation, causation, certainty, and tentativeness, and perception categories comprising of see, hear, feel, percept, insight, and relative; and (3) linguistic style attributes, including lexical density (verbs, auxiliary verbs, adverbs, prepositions, conjunctions, articles, inclusive, and exclusive), temporal references (past, present, and future tenses), social/personal concerns (family, friends, social, work, health, humans, religion, bio, body, money, achievement, home, sexual, and death), and interpersonal awareness and focus (first-person singular, first-person plural, and second-person and third-person pronouns). Each tweet was represented as a vector of normalized LIWC scores for each of the preceding 50 categories.

Thus, the feature space for the classifier was 550; 500 *n*-grams and 50 LIWC categories.

##### Feature Selection to Improve Predictive Power

As the linguistic attributes of text contain several correlated features, the classification model tends to be unstable. To improve the predictive power of the model, feature scaling and feature selection methods were employed. First, feature scaling was used to standardize the range of features. The LIWC features were within a normalized range of 0 to1; however, the *n-* gram features represented frequency counts that required standardization. The min-max rescaling technique was used to scale the *n-* gram features to the range of 0 to1. This technique scales a feature vector “x” by converting it to the ratio of difference between x and min(x), and difference between max(x) and min(x), where min(x) and max(x) represent the minimum and maximum value of all values in the vector x.

Next, feature selection was used to eliminate noisy features, which identifies the most salient variables used to predict the outcome. Specifically, the filter method was used where features are selected on the basis of their scores in various statistical tests for their correlation with the outcome variable. Adopting the ANOVA *F* test reduced the feature space from 550 features to *k* –best features (where *k*=350) by removing noisy and redundant features.

##### Classification Algorithm

Finally, training data represented by the top *k* features was fed into a model to learn the classification task. The model was trained over several algorithms including the Gaussian naïve Bayes, random forest, logistic regression, and support vector machines [25]. Among these, the best performing algorithm on cross-validation was used for analysis.

## Results

### Linguistic Characteristics

Table 2 represents comparison data between users with schizophrenia disclosures deemed genuine and the control cohort. Significance using the Mann-Whitney *U* test for all 50 LIWC categories are reported as well as the relative difference in means.

### Results of Machine Learning Classification

**Figure 1:**
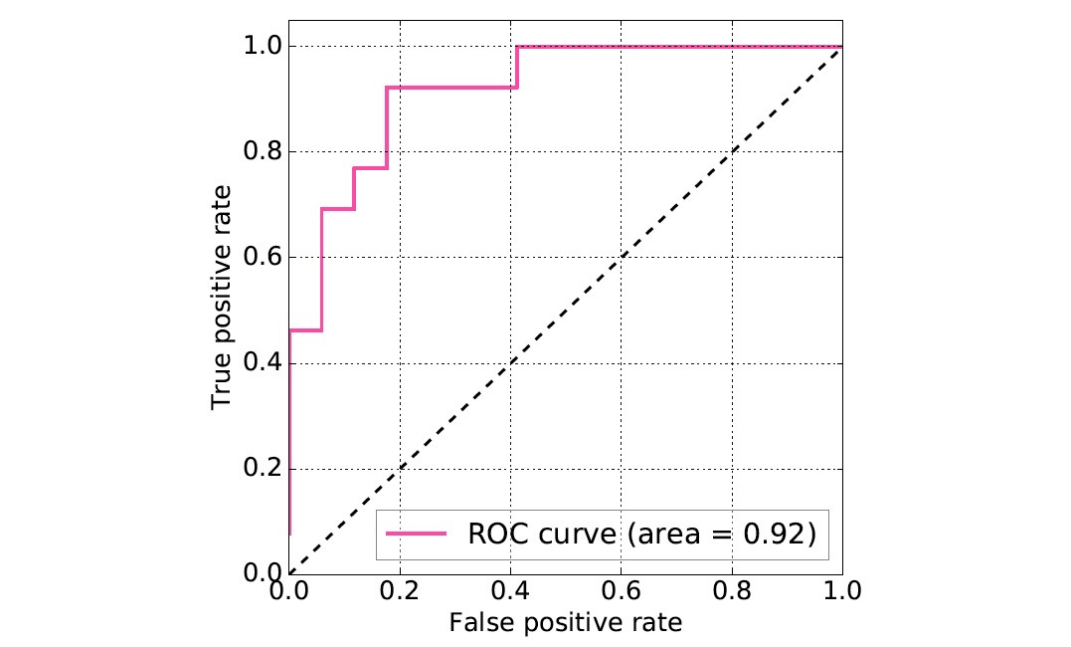
Receiver operating characteristic (ROC) curves for the classification task.

To evaluate the performance of the classification model, a 10-fold cross-validation method was used. During each fold (iteration), the data was split into a 70% training set and 30% validation set. A model was then constructed on the 70% data and tested on the remaining 30%. Among the several classification algorithms that were applied, a random forest performed best with an average receiver operating characteristic (ROC) area under the curve (AUC) score of 0.88. The best performance for the classifier was 0.95 by the same AUC metric (see Table 3). The ROC curve is presented in Figure 1.

**Table 2:** Mann-Whitney *U* test results comparing the linguistic differences between users with schizophrenia and the control datasets.

LIWC category		Difference in mean LIWC scores between groups	*U* stat	*P*^a^
**Affective attributes**				
	Positive affect	0.262	8517.5	.002
	Negative affect	0.283	7873.5	<.001
	Sadness	0.241	5301.5	<.001
	Swear	0.164	8557.5	.002
**Lexical density**				
	Auxiliary verbs	0.319	5712.5	<.001
	Preposition	0.186	7162.0	<.001
	Article	0.426	5812.0	<.001
	Inclusive	0.410	8262.5	<.001
	Exclusive	0.347	4753.0	<.001
	Quantifier	0.079	991.0	<.001
**Temporal references**				
	Past tense	0.194	7809.5	<.001
	Present tense	0.304	7501.0	<.001
	Future tense	0.185	4130.5	<.001
**Interpersonal awareness and focus**				
	First-person singular	0.024	3387.0	<.001
	First-person plural	0.006	8401.5	<.001
	Third person	0.243	7329.5	<.001
	Indefinite pronoun	0.265	2691.5	<.001
**Cognition and perception attributes**				
	Cognitive mechanisms	0.307	9418.0	.04
	Discrepancies	0.220	8975.5	.01
	Inhibition	0.257	7738.5	<.001
	Negation	0.187	9318.5	.03
	Causation	0.353	8023.5	<.001
	Certainty	0.110	6101.5	<.001
	Tentativeness	0.266	1841.5	<.001
	Hear	0.163	1796.5	<.001
	Feel	0.270	7555.5	<.001
	Perception	0.257	3340.5	<.001
	Insight	0.396	7918.5	<.001
**Social/Personal concerns**				
	Friends	–0.068	3269.0	<.001
	Work	0.036	5917.5	<.001
	Health	1.143	6775.0	<.001
	Humans	0.039	2963.5	<.001
	Biological Processes	0.427	7587.5	<.001
	Body	0.150	8021.5	<.001
	Achievement	0.087	6057.5	<.001
	Home	0.134	6261.5	<.001
	Sexual	0.494	8898.5	.007

^a^Based on Bonferroni correction.

**Table 3:** Classification results to distinguish between schizophrenia users and control users.

Results	Accuracy	Precision	Recall	F1 score	ROC AUC
Best performance	0.90	0.92	0.87	0.90	0.95
Average over 10 folds, mean (SD)	0.81 (0.07)	0.80 (0.09)	0.82 (0.05)	0.80 (0.07)	0.88 (0.04)

**Table 4:** Confusion matrix showing agreement and disagreement between the machine learning classifier and the experts.

Machine label	Expert annotation	
	Yes	No
Yes	14	37
No	4	45

### Verification in Unseen Data

To test the models for predicting new, unseen data, a sample of 100 users was passed through the classifier. The same sample was also provided to clinicians for appraisals. The confusion matrix displaying agreement between the two labels (machine and expert) is presented in Table 4.

By taking the expert annotations as true outcome and the machine labels as predicted outcome, true positive, true negative, false positive, and false negative scores were computed. Precision (positive predictive value) was calculated using true positive/(true positive+false positive) and recall (sensitivity) was calculated using true positive/(true positive+false negative). Accuracy (specificity) was calculated by the proportion of true results (both true positive and true negative) among the total number of cases examined (true positive+true negative)/(true positive+true negative+false positive+false negative). The resulting precision, recall, and accuracy measures were 0.27, 0.77, and 0.59, respectively.

## Discussion

### Main Findings

These data contribute to a growing body of literature using language to automatically identify individuals online who may be experiencing mental illness, including depression [16,22,27], postpartum mood disorders [28], suicide [29], posttraumatic stress disorder [30], and bipolar disorder [23]. To date, the majority of studies have used a computational approach to flag publicly available social media profiles of users who self-disclose with limited input from mental health clinicians to assess the authenticity of online disclosure. In this study, expert appraisal eliminated more than 70% of Twitter profiles that might have otherwise been recognized by computerized models as belonging to users with schizophrenia. These data reinforce the need for ongoing collaborations integrating expertise from multiple fields to strengthen our ability to accurately identify and effectively engage individuals with mental illness online. These collaborations are crucial to overcome some of mental illnesses’ biggest challenges using digital technology.

A major challenge in treating schizophrenia remains the lengthy delay between symptom onset and receiving appropriate care. Results from the Recovery After Initial Schizophrenia Episode-Early Treatment Program (RAISE-ETP) trial [31] suggest that the median duration of untreated psychosis is 74 weeks [32] and support the established hypothesis that lengthy duration of untreated psychosis (DUP) leads to worse outcomes [31,33]. At the same time, there is compelling evidence to suggest that linguistic and behavioral changes manifest on the pages of social media before they are clinically detected, providing the prospect for earlier intervention [22,28,34]. As more and more individuals are regularly engaging with digital resources, researchers must explore novel and effective ways of incorporating technological tools into DUP reduction strategies. Identifying linguistic signals of psychosis online might be an important next step to facilitate timely treatment initiation.

Once identified, social media provides an unparalleled opportunity to explore various engagement strategies. Recently, Birnbaum et al [35] used Google AdWords to explore aspects of digital advertising most effective at engaging individuals online. Digital ads were shown to be a reasonable and cost-effective method to reach individuals searching for behavioral health information. Similar strategies could be employed to engage users via social media platforms identified as potentially experiencing schizophrenia. These strategies would require careful consideration because there is a delicate line between overintrusiveness and concern. More research is needed to better define the trajectory between online activity and making first clinical contact to explore opportunities for digital intervention. Additionally, the ethical and clinical implications of identifying markers of mental illness online require thorough and careful evaluation. Existing ethical principles do not sufficiently guide researchers conducting social media research. Furthermore, new technological approaches to illness identification and symptom tracking will likely result in a redefinition of existing clinical rules and regulations. Although the potential beneficial impact of social media integration could be transformative, new critical questions regarding clinical expectations and responsibilities will require resolution.

The degree of agreement between the classifier and the experts in this study suggests that the classifier performs well at eliminating inauthentic noisy samples, but was overinclusive in labeling true cases of schizophrenia. For example, although the post “My parents are convinced I have schizophrenia,” was labeled by the classifier as a genuine disclosure, clinicians deemed it to be a noisy sample, reflecting a more careful and conservative approach. Therefore, the classifier can theoretically assist in triaging massive amounts of digital data to provide cleaner samples to experts who can then gauge the authenticity of the disclosure.

### Comparison With Prior Work

Consistent with prior trials [11-15,18,36], first-person pronouns were found to be significantly increased in the psychosis group, suggesting greater interpersonal focus. Additionally, these data replicate findings that biological processes, including words such as “body” and “health,” are more frequently used in psychosis [17], suggesting a greater awareness or focus on health status. Furthermore, the psychosis group was significantly less likely to use words from the “friends” category, possibly associated with social withdrawal. Although language dysfunction, and specifically thought disorder, is an established core symptom of schizophrenia, these data suggest that subtle, more granular changes may additionally be associated with schizophrenia. Furthermore, these data suggest that changes can be detected online, reinforcing exploration of novel Internet-based early identification strategies.

### Limitations

Confirming a diagnosis of schizophrenia via Twitter disclosure remains impossible without access to the psychiatric histories of those self-disclosing. Additionally, although some individuals may have psychotic symptoms (in the context of severe depression or mania), they may not meet full diagnostic criteria for schizophrenia. Exploring tweets surrounding the disclosure, taking a deeper look at an individual’s profile, and implementing expert consensus certainly improved diagnostic accuracy. Secondly, the research team only had access to publicly available Twitter profiles. It is likely that many individuals who chose to self-disclose online prefer to keep their profiles private and only accessible to select individuals. Many individuals with schizophrenia chose not to self-disclose via social media at all and therefore would not have been identified in this project. To overcome these challenges, we have begun extracting social media data from consenting individuals with known clinical diagnoses of schizophrenia, allowing for exploration of online markers of psychosis from individuals who might not otherwise have publically available data. Additionally, the current classifier was developed using exclusively linguistic variables. Future work must consider incorporating nonlinguistic data including frequency and timing of posts, changes in level of activity, and social engagement online. Finally, these findings may be limited to Twitter users, who may differ from individuals who use other platforms or may use Twitter differently from other sites.

### Conclusion

Existing online resources may be capable of sensing changes associated with mental illness offering the prospect for real-time objective identification and monitoring of patients. Ongoing multidisciplinary collaborations are crucial to perfect detection and monitoring capabilities for complex mental illnesses such as schizophrenia. To ensure effective incorporation of digital technology into early psychosis intervention, further research must explore precisely how symptoms of mental illness manifest online through changing patterns of language and activity as well as palatable, respectful, and effective treatment and engagement strategies once an individual is identified online.

## Abbreviations

AUC: area under the curve
DUP: duration of untreated psychosis
LIWC: language inquiry word count
RAISE-ETP: Recovery After an Initial Schizophrenia Episode-Early Treatment Program
ROC: receiver operating characteristic
tf-idf: term frequency-inverted document frequency
